# Echocardiographic Assessment of Pulmonary Arterial Hypertension for Pediatricians and Neonatologists

**DOI:** 10.3389/fped.2017.00168

**Published:** 2017-09-04

**Authors:** Gregory James Skinner

**Affiliations:** ^1^East Midlands Congenital Heart Centre, University Hospitals of Leicester NHS Trust, Glenfield Hospital, Leicester, United Kingdom

**Keywords:** pulmonary hypertension, pulmonary vascular resistance, echocardiography, pediatrics, neonatology

## Abstract

There is a growing awareness of the role that increased pulmonary vascular resistance (PVR) plays in many pathologies; therefore, assessment of pulmonary artery pressure (PAP) is an increasingly requested investigation in the critical care environment. This article will go through the basic concepts regarding PAP and PVR, then will go on to outline the various echocardiographic parameters which are used to assess them. Finally, an outline of how to undertake this assessment will be presented.

## Basic Concepts and Definitions

To start with, it is very important to understand the differences and interdependencies of three commonly quoted (and confused) variables (Table 1).

**Table 1:** Important variables in the assessment of pulmonary vascular resistance.

Variable	Units of measurement
Flow	L/min (indexed to body surface area as L/min/m^2^) notation: “Q”
Resistance	Woods unit·m^2^ (WU·m^2^)
Pressure	Torr (mmHg)

Pressure is the product of flow and resistance and is related to flow and resistance by the hydraulic Ohm’s law analogy:

(1) Pressure=Flow×Resistance

In order to account for the pressure distal to the intrapulmonary vasculature, we need to add in the effect of left atrial pressure (LAp):

(2) $$\begin{array}{l} \text{PAP}=\text{Qp}\times \text{PVR}+\text{LAp}\\ ∴\text{PVR}=\frac{\text{PAP}-\text{LAp}}{\text{Qp}} \end{array}$$

*PAP, pulmonary artery pressure; Qp, pulmonary artery blood flow; PVR, pulmonary vascular resistance; LAp, left atrial pressure*.

The gold standard for measurement of pulmonary vascular resistance (PVR) is cardiac catheterization (1–3), as this can measure all of these variables: pulmonary artery pressure (PAP), LAp (or pulmonary capillary wedge pressure), and Qp (Fick method or thermodilution). This is obviously invasive and not reproducible at the bed-side.

In most critical care patients in whom there is a concern about “pulmonary hypertension,” the underlying pathological process of interest is increased PVR. We cannot directly measure PVR on echocardiography, so we have to use another measurement as a surrogate for it: i.e., PAP. Unfortunately, there are other pathological processes which affect this; therefore, it is always vital to remember that:

(3) PVR≠PAP

Looking at Eq. 2, we can determine that there are four mechanisms, which will lead to increased PAP:

(1) Increased PVR.
(2) Increased Qp.
(3) Increased LAp.
(4) Any combination of the above.

Any assessment of PVR requires the assessment of all of these possibilities. Pulmonary arterial hypertension (PAH) from increased PVR can only be assumed if there is no increased flow (e.g. left-to-right shunt) and/or there is no suggestion of increased LAp (e.g. due to left ventricular failure or mitral stenosis).

## Quantification

In adults, pulmonary hypertension is defined as a mean PAP > 25 mmHg (4). This can be derived from the systolic PAP (5):

(4) mean PAP≅(0.61×systolic PAP)+2

In adult practice there are well-defined thresholds for indicating the degree of PAH (see Table 2). In children (and especially neonates and infants), the normal PVR (and, therefore, PAP) changes with age. To quantify the degree of pulmonary hypertension, therefore, the pulmonary artery peak systolic pressure is often quoted as a fraction of the systemic systolic blood pressure (Table 3).

**Table 2:** Quantification of PAH in adults.

Mean pulmonary artery pressure (PAP)	(≈Systolic PAP)	Degree of PAH
<25 mmHg^a^	<40 mmHg	Normal
25–40 mmHg	40–60 mmHg	Mild
41–55 mmHg	60–90 mmHg	Moderate
>55 mmHg	>90 mmHg	Severe

*^a^Normal mean PAP is defined as <20 mmHg, but PAH is defined as mean PAP ≥25 mmHg. Patients with a mean PAP of 21–24 mmHg (≈systolic PAP 30–40 mmHg) should be considered at risk of developing PAH, and followed up (4)*.

**Table 3:** Quantification of PAH in children.^a^

Systolic PA pressure/systemic systolic BP	Degree of PAH
<1/3	Normal
1/3–2/3	Mild
2/3–1	Moderate
>1	Severe

*^a^These values are not standard, but are frequently used in clinical practice*.

## Echocardiographic Measurement of PAP

### Tricuspid Regurgitation (TR)

The most common method used for assessing PAP is to perform a continuous wave spectral Doppler analysis of the TR jet (6, 7). Most patients will have a degree of TR, which is often (though not always) made worse when the right ventricle is operating at higher pressures.

The modified Bernoulli equation states that the pressure gradient between either side of a fixed obstruction with no significant length, is proportional to the velocity (*v*) of the flow across that obstruction:

(5) $$\text{Pressure gradient}=4\upsilon^{2}$$

Measuring the velocity of the TR jet can, therefore, give an estimate of the pressure gradient between the right ventricle and right atrium at peak systole. Therefore:

(6) RVSP=TR max PG+mean RAp

*RVSP, right ventricular systolic pressure; TR max PG, Bernoulli equation derived pressure gradient from the peak tricuspid regurgitation velocity; RAp, right atrial pressure*.

Central venous pressure monitoring is often used in the critical care environment, and can usually be treated as analogous to the right atrial pressure. If this is not available, the RAp component is often ignored, as its value usually only contributes a small amount to the overall result (especially in the context of increased RVSP).

#### Pitfalls

**Figure 1:**
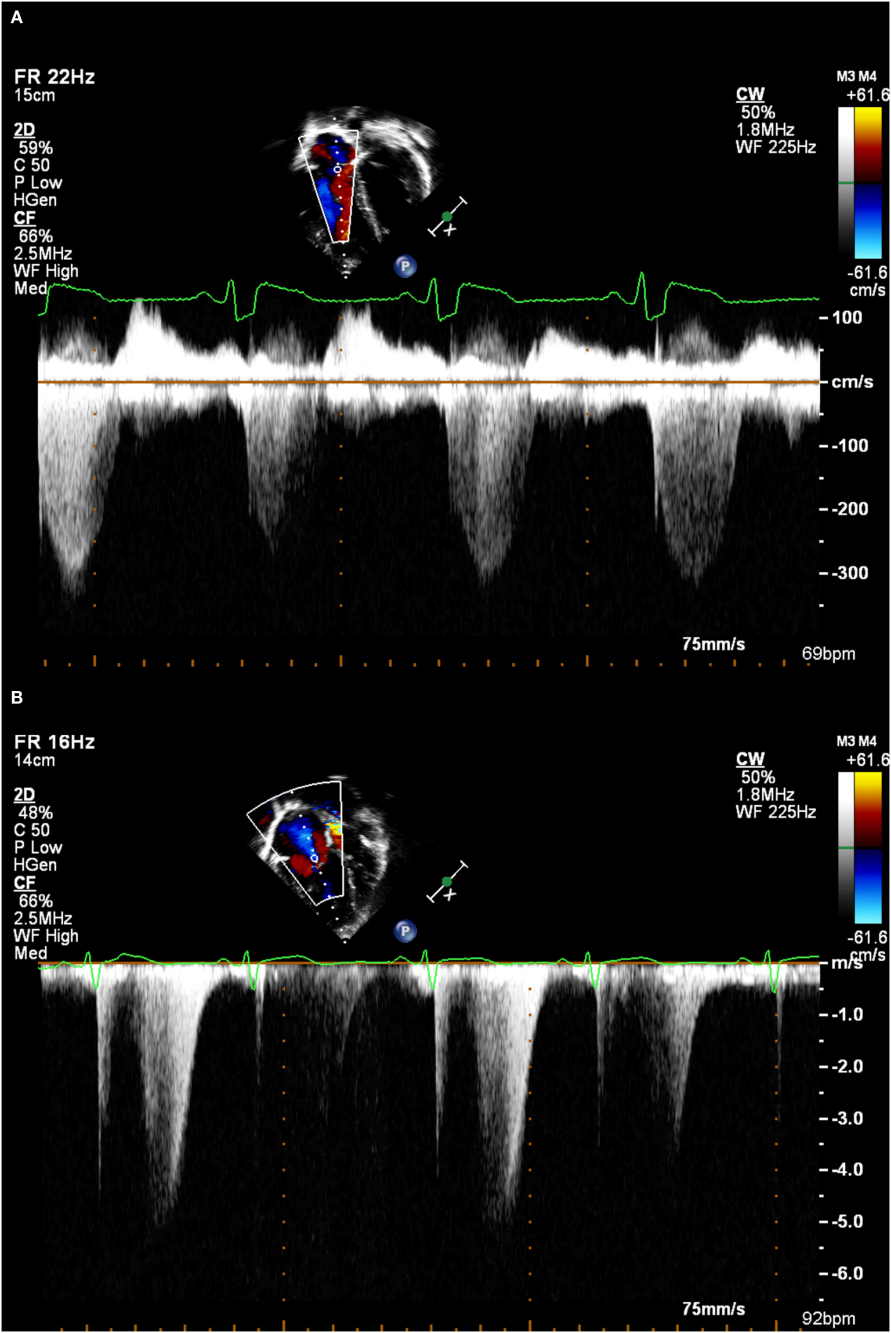
**(A)** A good tricuspid regurgitation jet envelope, suitable for analysis (note incomplete envelope on second beat due to respiratory motion). **(B)** An incomplete tricuspid regurgitation jet envelope, which will underestimate right ventricular systolic pressure.

- Many patients will have no or only trivial TR. Given the motion of the AV valve plane during systole, it can be impossible to obtain a spectral Doppler TR trace for the duration of systole. The spectral Doppler trace of TR is quite characteristic, and values should not be quoted unless there is a clear trace, otherwise, there is the risk of a significant underestimate. This means that this technique cannot be used for all patients (see Figures 1A,B).
- The Doppler interrogation beam should be within 20° of the jet direction, or there will be significant underestimation of the velocity. This is not always possible with some eccentric jets.
- Many confuse the quantity of TR with the pressure gradient. It is possible to have severe TR with a low velocity or mild TR with a high velocity. The two are not directly related.
- In severe TR, the right atrial pressure will increase rapidly during systole, resulting in an underestimation of the RVSP.
- If there is any right ventricular outflow obstruction (e.g., pulmonary stenosis), then it is important to remember that the RVSP will not reflect the systolic PAP. It is very important to check the whole of the right ventricular outflow (subpulmonary area, pulmonary valve, main and branch pulmonary arteries) for evidence of obstruction.
- Large left-to-right shunts [e.g., large ventricular septal defect (VSD), aortopulmonary window, PDA] will cause equalization of right ventricular/PAP with left ventricular/aortic pressure. There is little point in measuring the TR velocity in this context as it will just reflect the systemic systolic blood pressure.
- A VSD ejecting into the region of the tricuspid valve may “contaminate” the spectral Doppler trace, making it uninterpretable.

### Pulmonary Regurgitation (PR)

**Figure 2:**
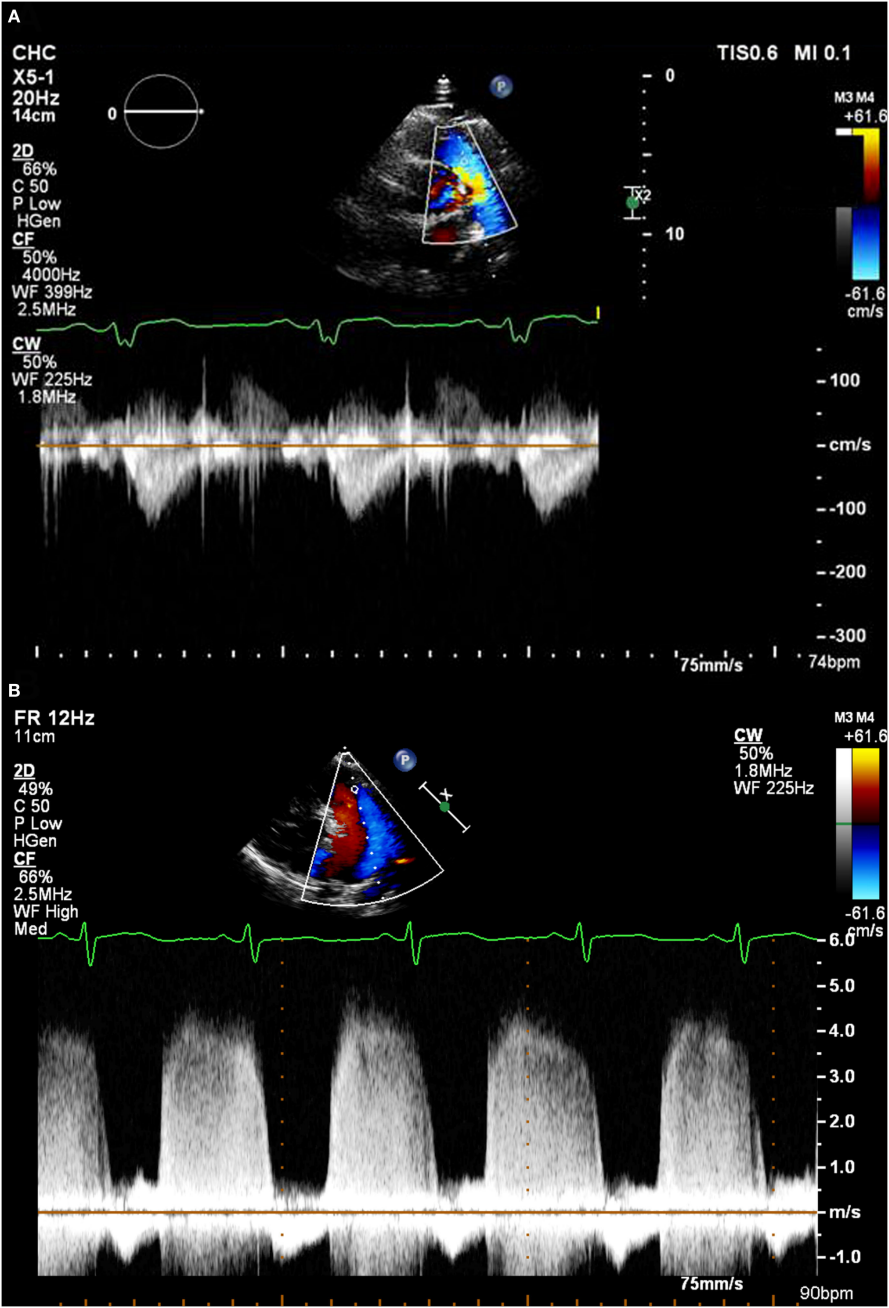
**(A)** A pulmonary regurgitation envelope with normal pulmonary artery pressure. **(B)** A pulmonary regurgitation envelope in a patient with very high pulmonary artery pressure.

Similar to TR, most (but not all) patients will have some degree of PR. Analysis of the spectral Doppler pattern from this can also give important information regarding the PAP (8–10) (see Figures 2A,B). The following values can be obtained from this:

(7) $$\begin{array}{l} \text{Mean PAP}\approx \text{PR max PG}+\text{RAp}\\ \text{Diastolic PAP}=\text{PR EDPG}+\text{RAp} \end{array}$$

*PAP, pulmonary artery pressure; PR max PG, Bernoulli equation derived pressure gradient from the peak pulmonary regurgitation velocity; RAp, right atrial pressure; PR EDPG, Bernoulli equation derived pressure gradient from pulmonary regurgitation velocity at end diastole*.

#### Pitfalls

- Not all patients will have a PR jet.
- It is vitally important to capture the full spectral Doppler envelope and be well aligned with it.
- This technique is not reliable in the context of severe PR.
- Given that the magnitude of the mean and diastolic PAP is less than the RVSP/systolic PAP, the RAp has more of an influence on this measurement. It is, therefore, more prone to underestimation if this is not known.

## Assessment of Interventricular Septum

**Figure 3:**
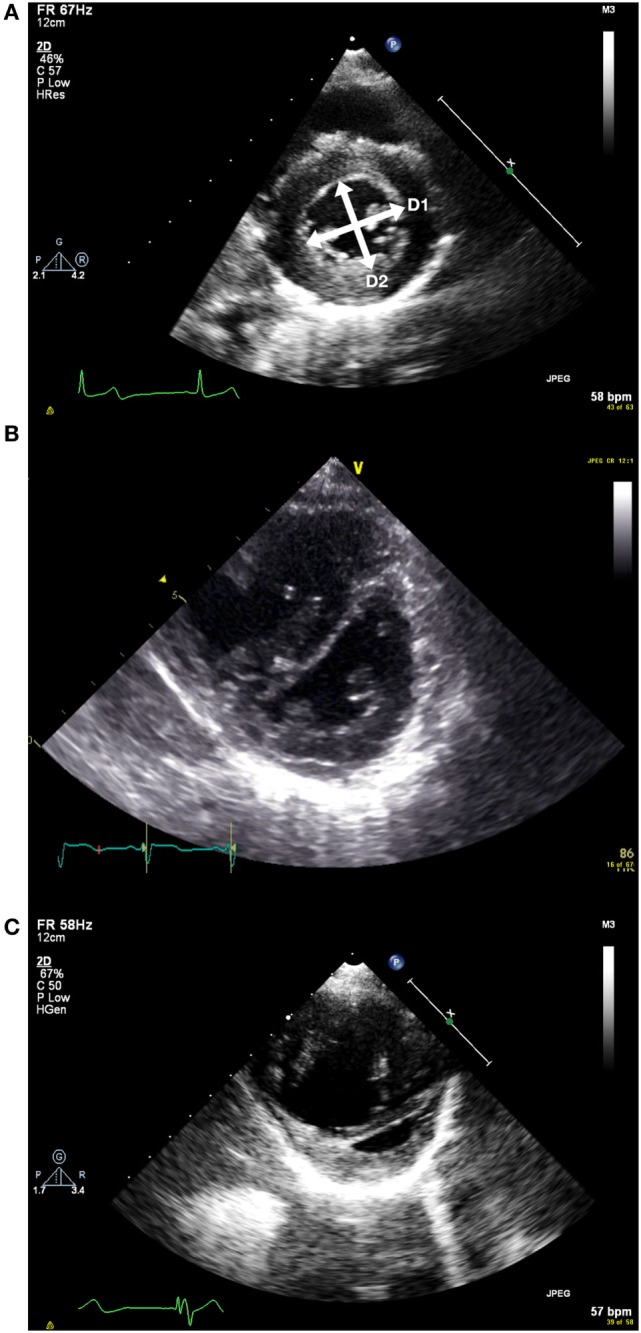
**(A)** Short axis view of the left ventricle in end systole. D1 and D2 represent the measurements taken to measure the end systolic eccentricity index. This shows a patient with normal right ventricular systolic pressure. **(B)** Flattened septum with increased end systolic eccentricity index with moderately increased right ventricular systolic pressure. **(C)** Short axis view with severely increased right ventricular systolic pressure. Note RV dilatation.

The geometry of the interventricular septum is dependent on the relative pressures within the ventricles. Usually, as the left ventricle is at much higher pressure than the right ventricle, the interventricular septum is pushed rightwards. This gives the characteristic circular profile of the left ventricle on the short-axis view. As right ventricular pressure increases, the septum is pushed more toward the left resulting in the left ventricle becoming “*D*-shaped.” While this does not provide quantitative information, it allows assessment in patients regardless of whether they have TR, PR, or a shunt (see Figures 3A–C).

The end systolic eccentricity index (EIs) allows some categorization of the RV pressure (11) (see Table 4). It is measured on the short-axis view of the left ventricle, just below the level of the mitral valve tips. At end systole (i.e., when the LV cavity is smallest), the diameter of the left ventricle is measured parallel to (D1) and perpendicular to (D2) the interventricular septum.

(8) EIs=D1/D2

**Table 4:** Categorisation of RV pressure based on EIs (11).

End systolic eccentricity index	RV pressure
<1.15	Normal/mildly increased
1.15–1.29	Moderately increased
≥1.3	Severely increased

*EIs, end systolic eccentricity index; D1, LV diameter parallel to interventricular septum; D2, LV diameter perpendicular to interventricular septum*.

Serial measurement of the EIs over time may allow monitoring of the progression of the RV pressure.

### Pitfalls

- Interventricular conduction abnormalities (i.e., left or right bundle branch block) will alter the relative timing of peak left and right ventricular pressure, making this method unreliable.
- The presence of a large VSD will affect the motion of the interventricular septum, making this method unusable.
- Given this method indirectly measures the *difference* between LV and RV pressure, anything which increases LV pressure (e.g., aortic stenosis, coarctation of the aorta) will result in an underestimate of RV pressure.

## Shunts

The presence of a shunt between the systemic and pulmonary circulations also allows estimation of the relative pressures of the two systems. Please note that pre-tricuspid valve level shunts (e.g., systemic arteriovenous malformation, partial anomalous pulmonary venous connection, atrial septal defect) do not communicate between the high-pressure portions of the circulation, so the points below do not apply.

### Large Shunts

As previously stated, large non-restrictive shunts (in the absence of any outflow tract stenosis) will result in equalization of the systemic and pulmonary pressures. The direction and quantity of flow across the shunt will reflect the relative difference between the systemic vascular resistance and PVR—this can be assessed clinically as well as on echocardiography (NB: here, we are evaluating flow as opposed to pressure, see Table 5).

**Table 5:** Evaluation of PVR in the presence of a large shunt.

Shunt direction on echo	Clinical findings	Pulmonary vascular resistance (PVR) vs SVR
Left-to-right	Normal saturations Respiratory distress Pulmonary congestion Hepatomegaly Heart failure	PVR < SVR
No net shunt (bidirectional)	Normal/variable saturations (may have labile PVR) Asymptomatic from shunt	PVR ≈ SVR
Right-to-left shunt	Low saturations (if PDA, then may have differential upper/lower limb saturations) Difficult to oxygenate Asymptomatic from shunt	PVR > SVR

### Small Shunts

If the size of the shunt is small, then it will not greatly affect the pressures between the two sides of the circulation. Therefore, using the modified Bernoulli equation will again allow estimation of the relative difference between the systemic and pulmonary pressures.

For example, if there is a small VSD, the peak systolic velocity across it will reflect the pressure gradient between the ventricles at peak systole. Therefore:

(9) RVSP=Systemic systolic BP−VSD max PG

*RVSP, right ventricular systolic pressure; BP, blood pressure; VSD max PG, Bernoulli derived pressure gradient from the peak ventricular septal defect velocity*.

### PDA Flow

The above principle applies to a PDA, although this also gives information regarding the differential between the relative diastolic pressures as well. Given that the diastolic PAP is less influenced by flow, it is often thought to better reflect the PVR and is less affected by shunts. Patterns of ductal flow can be used to infer PVR status (see Table 6).

**Table 6:** Evaluation of pulmonary artery pressure based on PDA spectral Doppler pattern.

Spectral Doppler trace (blue = right-to-left flow, red = left-to-right flow)	Comment
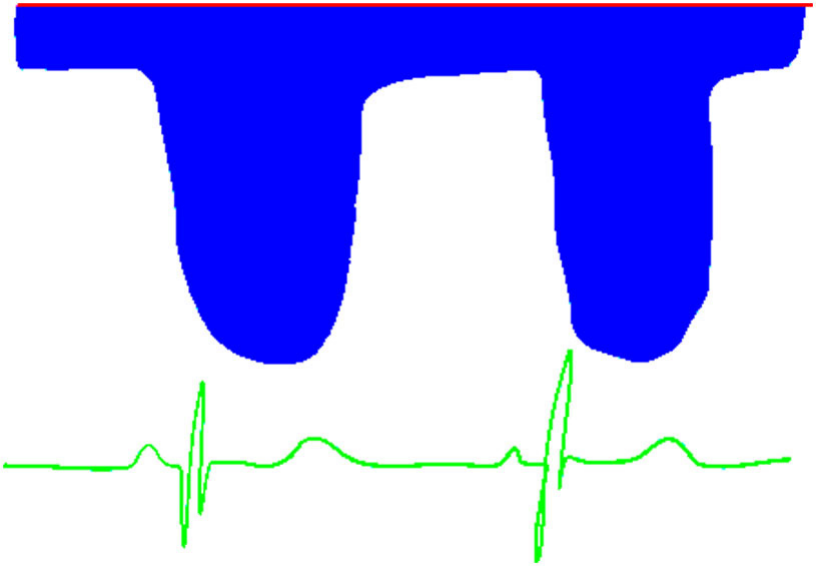	Right-to-left shunt in systole and diastole Pathological trace—indicates very elevated pulmonary artery pressure Likely to have differential upper and lower limb saturations
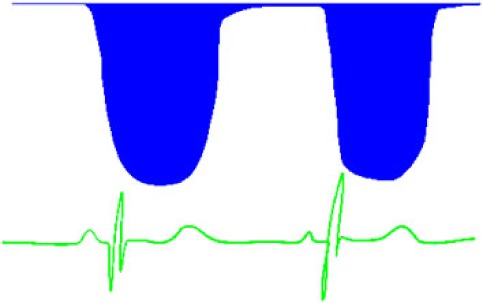	Right-to-left shunt in systole, no shunt in diastole Indicates significantly elevated pulmonary artery pressure
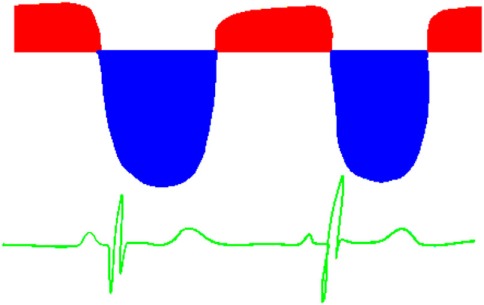	Right-to-left shunt in systole, left-to-right shunt in diastole Systolic pulmonary artery pressure (PAP) > systolic BP Normal trace in newborns [physiologically high pulmonary vascular resistance (PVR)] May reflect increased PVR or large shunt
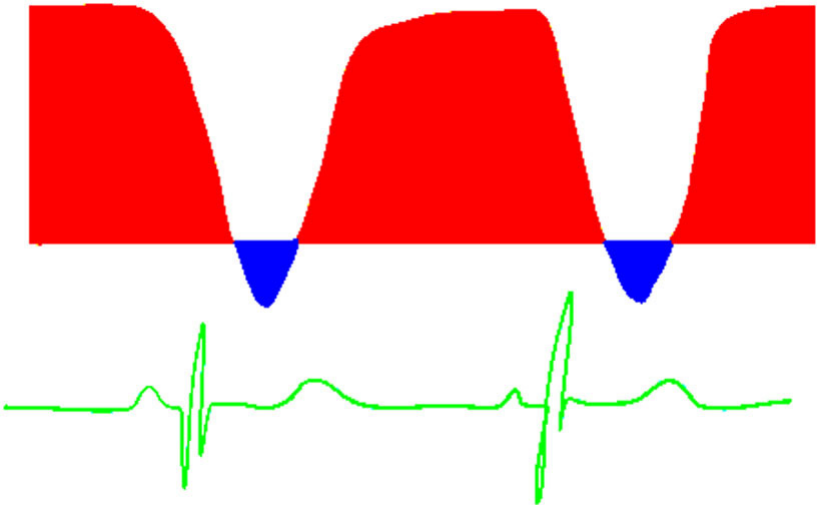	Shunt predominantly left-to-right, some right-to-left flow in systole Transitional trace in newborn, indicating falling PVR Indicates elevated systolic PAP
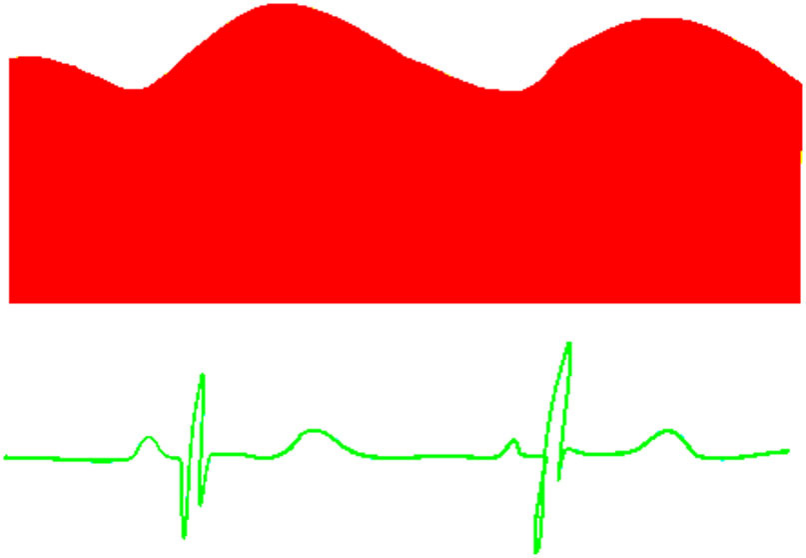	Continuous left-to-right shunt Systolic and diastolic PAP < systolic and diastolic systemic BP Peak velocity will reflect difference between systolic BP and systolic PAP

Given the high incidence of PDA in the neonatal population, this is often the best way of assessing PAP in this group.

## Putting it all Together

**Figure 4:**
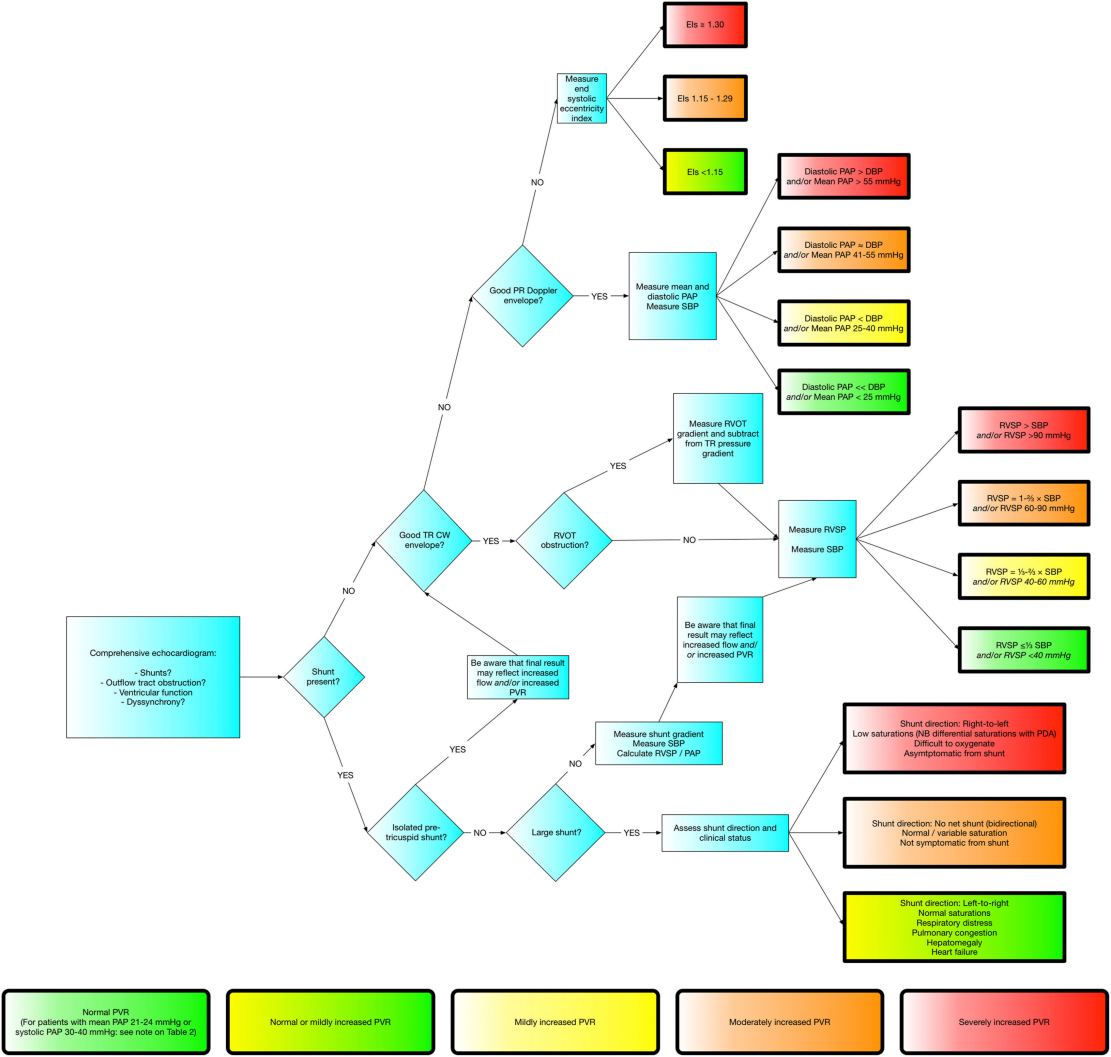
Flowchart demonstrating approach to evaluating pulmonary hypertension.

There is no one perfect method of assessing PAP or PVR. Figure 4 demonstrates a framework built upon the methods stated above which should work for the vast majority of patients. It is always important to remember the pitfalls of all of these methods and to go back to first principals if the information obtained does not fit with the clinical picture.

Pulmonary hypertension and/or elevated PVR is a complicated area, with highly specialized knowledge required to effectively diagnose or manage. The above guide provides a framework to allow the pediatric or neonatal echocardiographer to suspect the presence of these pathologies; but it would be highly recommended that if they are suspected, then advice from a specialist should be sought.

## Author Contributions

The author confirms being the sole contributor of this work and approved it for publication.

## Conflict of Interest Statement

The author declares that the research was conducted in the absence of any commercial or financial relationships that could be construed as a potential conflict of interest.
